# In the eye of the ophthalmologist: the corneal microbiome in microbial keratitis

**DOI:** 10.1007/s00417-023-06310-y

**Published:** 2023-11-23

**Authors:** Susanna Sagerfors, Sofie Edslev, Birgitta Ejdervik Lindblad, Berit Lilje, Marc Stegger, Bo Söderquist

**Affiliations:** 1 Department of Ophthalmology, Faculty of Medicine and Health, Örebro University, SE 701 82 Örebro, Sweden; 2 Department of Bacteria, Parasites and Fungi, Statens Serum Institut, Copenhagen, Denmark; 3 School of Medical Sciences, Faculty of Medicine and Health, Örebro University, SE 701 82 Örebro, Sweden; 4 Antimicrobial Resistance and Infectious Diseases Laboratory, Harry Butler Institute, Murdoch University, Murdoch, Australia

**Keywords:** Keratitis, Corneal microbiome, Sequencing, Indirect inoculation

## Abstract

**Purpose:**

To describe the bacterial findings by a targeted sequencing approach from corneal samples of patients with microbial keratitis and factors influencing culture outcome of indirectly inoculated corneal specimen.

**Methods:**

Prospective inclusion of patients fulfilling predefined criteria of microbial keratitis. Samples from the corneal lesion were collected and dispensed in liquid transport medium, from which both culture and targeted amplification and sequencing of the V3-V4 region of the 16S rRNA gene were carried out. Additional standard corneal culture from the corneal lesions was also performed. Factors influencing culture outcome of indirectly inoculated corneal samples were identified by a multivariate regression model incorporating quantitative data from sequencing.

**Results:**

Among the 94 included patients with microbial keratitis, contact lens wear (*n* = 69; 73%) was the most common risk factor. Contact lens wearers displayed significant differences in the bacterial community composition of the corneal lesion compared to no lens wearers, with higher abundance of *Staphylococcus* spp., *Corynebacterium* spp., and *Stenotrophomonas maltophilia*. Targeted sequencing detected a potential corneal pathogen in the highest proportional abundance among 9 of the 24 (38%) culture-negative patients with microbial keratitis. Age, bacterial density in the sample, and prior antibiotic treatment significantly influenced culture outcome of indirectly inoculated corneal samples.

**Conclusion:**

Targeted sequencing may provide insights on pathogens in both culture negative episodes of microbial keratitis and among subgroups of patients with microbial keratitis as well as factors influencing culture outcome of indirectly inoculated corneal samples.

**Supplementary Information:**

The online version contains supplementary material available at 10.1007/s00417-023-06310-y.


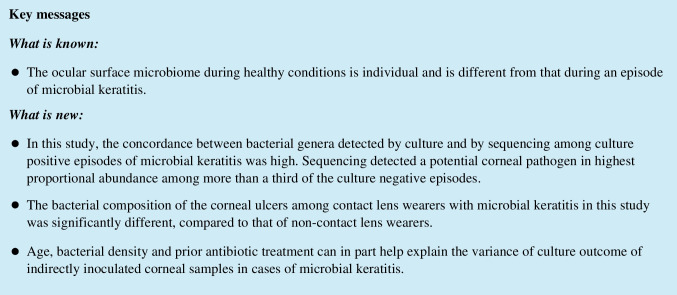


## Introduction

Microbial keratitis caused by bacteria, fungi, or protozoa is a common ophthalmological emergency that can result in visual impairment and blindness. Bacteria are the most common cause of microbial keratitis in Europe, North America, and Australia/Oceania, being reported in 88.3–98.3% of the culture-positive cases that coincides with a high prevalence of contact lens wear [1].

The clinical presentation is usually sufficient to raise suspicion of an infectious origin. Still detection and susceptibility testing of the causative agent are vital in the management and proper treatment of the disease. Corneal culture and direct microscopy with staining have been the gold standard in the diagnostic procedure of microbial keratitis [2, 3]. However, the practice of corneal cultures has limitations. Different microorganisms require distinct substrate and incubation conditions, and only a modest proportion of cases with a clinical suspicion of microbial keratitis are culture-positive, with studies showing a median of 50% and range of 32.6–79.4% [1].

High-throughput sequencing of bacterial DNA extracted from primary samples is an attractive approach to identifying bacteria within corneal samples of patients with microbial keratitis [4, 5]. One common approach is targeted amplicon sequencing of the bacterial 16S rRNA gene present in all bacteria. This gene contains regions with high sequence diversity between bacterial families, which allows the classification of bacteria, often at genus level and sometimes at species level. Detection and classification of common bacteria at species level can also be achieved with metagenomic shotgun sequencing, but at a much higher financial cost [6].

Previous sequence-based studies have shown that the conjunctival microbiome in the healthy eye is heterogeneous, almost individual, and influenced by both age and sex [7].

We have previously reported on microbial findings from two different culture-based approaches [8]. In the present study, we used a combined quantifiable targeted sequencing approach to (i) describe bacterial findings in relation to culture results and clinical parameters in corneal samples obtained from patients with microbial keratitis, (ii) explore presumptive pathogens in the subgroup of patients with microbial keratitis where corneal cultures, directly and indirectly inoculated, are negative, and (iii) investigate variables, such as bacterial density and clinical characteristics, that influence the culture outcome of indirectly inoculated corneal samples from patients with microbial keratitis.

## Methods

### Study population

This prospective study was approved by the Regional Ethical Review Board of Uppsala, Sweden (ref: 2018/120). Informed written consent was collected from all participants prior to inclusion. All aspects of the management of patients participating in this study and methods applied within this study were carried out in accordance with relevant guidelines and regulations and adhered to the ethical standards of the Declaration of Helsinki. All patients aged 18 or older presenting with an episode of suspected microbial keratitis (i.e., a corneal infiltrate with an overlying epithelial defect) at the Department of Ophthalmology, Örebro University Hospital, Sweden, between 10 September 2018 and 27 January 2020 were considered for inclusion [8].

Patients were included if the corneal culture from the infiltrate showed growth of bacteria, yeast, or protozoa (all growth was considered as significant) and/or the patient fulfilled at least one of the clinical criteria for microbial keratitis, that is, infiltrate within/overlapping the central 4 mm of the cornea and/or uveitis and/or pain [9]. The exclusion criterion was corneal sampling not performed according to the study protocol.

### Corneal culture according to two different approaches: standard culture and ESwab culture

The standard corneal culture procedure was in accordance with “Microbial keratitis Caused by Bacteria, Yeast, and Protozoa,” the Swedish state-of-the-art document on microbial keratitis during the study period. The corneal sampling order and culture procedure have been described previously [8]. In short, corneal samples were both directly inoculated (standard culture) and indirectly inoculated (ESwab culture) through liquid transport medium (modified Amies transport medium) of the ESwab kit (COPAN Italia S.p.A., Brescia, Italy) (see Supplementary Fig. 1). For details on culture media and incubation conditions, see Supplementary Table 1. The remaining transport medium of the ESwab kit, containing the corneal sample, was stored at − 80 °C pending further processing for DNA purification, PCR, quantitative PCR, and sequencing. Amplification and sequencing were performed after inclusion of the last patient in January 2020; hence the results of the targeted amplification and sequencing were not available during the ongoing disease episode.

Species determination was carried out with matrix assisted laser desorption/ionization time of flight-mass spectrometry (MALDI-TOF MS) with a Microflex LT and Biotyper 3.1 (Bruker Daltonik, Bremen, Germany).

### DNA extraction, targeted amplicon sequencing, and quantitative PCR

DNA extraction, 16S rRNA amplicon sequencing, and quantitative PCR were performed as previously described [10]. In short, bacterial DNA was extracted from 200 µl of the ESwab transport medium on a MagNA Pure 96 instrument using the DNA and Viral NA Small Volume Kit (Roche, Mannheim, Germany) with an enzymatic pre-lysis with 3 mg lysozyme, 4 units lysostaphin, and 25 units mutanolysin per sample (Sigma-Aldrich, Merck, Germany). Amplicon libraries were prepared via a two-step PCR amplification of the 16S rRNA V3–V4 regions [11] using primers with heterogeneity spacers to increase the sequence diversity. Amplicon libraries were sequenced using an Illumina v3 600-cycle reagent kit on a MiSeq instrument (Illumina Inc., San Diego, USA). The absolute abundance of bacteria was estimated by qPCR using the same primer regions as for the amplicon sequencing and a 16S-TQM-528R TaqMan probe [12]. Details of primers, sequence pre-processing and the PCR methodology are provided as supplementary material.

### Bioinformatics and statistics for the amplicon sequence data

Analyses of the amplicon sequence data were performed in R (v.4.0.2), using the following packages: DADA2 (v.1.12.1) [13] phyloseq (v.1.33.0) [14], vegan (v.2.5.6) [15], metacoder (v.0.3.5) [16], ANCOMBC (v.0.99.3) [17], and ggplot2 (v.3.3.2) [18]. The DADA2 package was used for determination of amplicon sequence variants (ASVs) and taxonomic classification using the Silva reference database (v.132) [19]. Sequence counts were agglomerated at genus level for all analyses, except when investigating bacterial richness (taxa counts) within samples, which was examined on the ASV level. Differences in richness between patient groups were tested with the Mann–Whitney *U* test. Differences in bacterial composition between selected patient groups (prior vs. no prior topical antibiotic treatment and contact lens wear vs. no contact lens wear) were studied by calculating sample-pairwise Bray–Curtis dissimilarities on Hellinger-transformed taxa counts followed by a statistical analysis of similarity (ANOSIM), where *R* (the effect size) close to 0 indicates completely similar compositions [20]. To compare differences in abundance of detected genera between contact lens wearers and non-wearers, a differential abundance analysis was performed using a composition of microbiomes with bias correction (ANCOM‐BC) modeling [17] with zero.cut set to 0.9 (exclusion of all genera present in less than 10% of the samples). The Benjamini‐Hochberg method was used to adjust for multiple testing. An adjusted *p*-value below 0.05 was considered to be of significance. The ANCOM-BC function was also used for testing whether any genera were more abundant within culture-negative samples than within culture-positive samples (culture-positive by either approach or both approaches). Here, zero.cut was set to 0.978, corresponding to the exclusion of genera present in less than two samples.

### Statistical analysis of the clinical data and regression model

The Mann–Whitney *U* test was used for comparisons of age, symptom duration, visual acuity, lesion size, and absolute quantity of bacterial DNA in the corneal samples. The χ^2^ test, or Fisher’s exact test when appropriate, was used for comparisons on sex, laterality, and risk factors for microbial keratitis (i.e., contact lens wear or not) and whether antibiotic treatment had preceded corneal culture. A logistic regression model was used to examine associations between predictive variables such as age, sex, laterality, and 16S rRNA gene copy number (log_10_units) [4] and prior antibiotic treatment and culture outcome from indirect inoculation of the ESwab sample. A quantile regression model was chosen to describe the absolute amount of 16S rRNA gene copy numbers, since heteroscedasticity of the residuals with respect to the outcome was detected. The statistical analysis was performed in SPSS (v. 25).

## Results

### Patient characteristics

In all, 110 episodes of microbial keratitis were considered for inclusion. Of these, 16 were excluded due to not fulfilling the age criteria (*n* = 2), failure to adhere to randomized sampling order (*n* = 2), lack of written consent (*n* = 1), loss of the stored ESwab sample intended for molecular analysis (*n* = 5), and very low sequence count (i.e. < 1000; *n* = 2). In addition, four patients had two episodes each, where the second episode for each of these four patients was excluded (*n* = 4). The remaining 94 episodes of microbial keratitis in 94 patients constituted the study population. In the quantitative analysis, quantitative data for five samples were not available.

Median age in the cohort was 44 years (range 18–84), 51/94 (54%) were men, and the right eye was affected in 47/94 (50%) of cases. Topical antibiotic treatment preceded corneal sampling in 16 patients. Supplementary Table 2 contains information on preceding topical antibiotics. The most commonly identified risk factor for microbial keratitis in the cohort was contact lens wear (73%; *n* = 69). The contact lens wearers were significantly younger than the non-wearers (median age 42 years vs. 51 years; *p* = 0.003). The episodes presumably caused by contact lens wear had a significantly shorter median duration of symptoms at first visit; two days compared to six days among the episodes with other risk factors (*p* < 0.001). In comparison to non-wearers, contact lens wearers also displayed a significantly higher median visual acuity (Snellen) at the first visit (1.0 vs. 0.5, *p* < 0.001) and smaller lesions (median longest diameter: 1.0 mm vs. 1.7 mm, *p* < 0.001) (Table 1).

**Table 1:** Background characteristics and clinical parameters of 94 patients with microbial keratitis in relation to culture outcome (total and divided by standard culture method vs. ESwab culture) and contact lens wear

	Total (*n* = 94)	Culture positive Total (*n* = 70) Culture positive Standard (*n* = 59) Culture positive ESwab (*n* = 54)	Culture negative Total (*n* = 24) Culture negative Standard (*n* = 35) Culture negative ESwab (*n* = 40)	*p*-value	Contact lens wear^a^ (*n* = 69)	No contact lens wear^b^ (*n* = 25)	*p*-value
Age in years, median (25th; 75th percentiles) [range]	44 (32; 55) [18–84]	44 (34; 56) [19–84] 45 (32; 57) [19–82] 45 (36; 62) [19–84]	41 (27; 53) [18–84] 41 (34; 52) [18–84] 40 (27; 48) [18–84]	0.435^ g^ 0.457^ g^ 0.032^ g^	42 (31; 50) [18–84]	51 (41; 72) [25–84]	0.003^ g^
Male sex, *n* (%)	51 (54%)	36 (51%) 34 (58%) 28 (52%)	15 (63%) 17 (59%) 2 (58%)	0.347^ h^ 0.394^ h^ 0.587^ h^	35 (51%)	16 (64%)	0.254^ h^
Right eye laterality, *n* (%)	47 (50%)	42 (60%) 35 (59%) 34 (63%)	5 (21%) 12 (34%) 13 (33%)	< 0.001^ h^ 0.019^ h^ 0.003^ h^	32 (46%)	15 (60%)	0.243^ h^
Contact lens wear ^a^, n (%)	69 (73%)	49 (70%) 39 (66%) 37 (69%)	20 (83%) 30 (86%) 32 (80%)	0.202^ h^ 0.037^ h^ 0.245^ h^			
Topical antibiotic treatment prior to sampling, n (%)	16 (17%)	8 (11%) 7 (12%) 7 (13%)	8 (33%) 9 (26%) 9 (23%)	0.025^i^ 0.084^ h^ 0.224^ h^	11 (16%)	5 (20%)	0.757^i^
Duration in days of symptoms ^c^ at first visit, median (25th; 75th percentiles) [range]	3 (1; 4) [0.5–61]	3 (1; 5) [1–31] 3. (1; 6) [1–31] 3 (1; 7) [1–31]	3 (2; 4) [1–61] 2 (1; 4) [1–61] 3 (1; 4) [1–61]	0.694^ g^ 0.131^ g^ 0.338^ g^	2 (1; 4) [1–61]	6 (3; 10) [1–31]	< 0.001^ g^
Snellen visual acuity at first visit^d^, median (25th; 75th percentiles) [range]	0.9 (0.4; 0.9) [amaurosis–1.0]	0.9 (0.3; 1.0) [amaurosis–1.0] 0.9 (0.3; 1.0) [amaurosis–1.0] 0.9 (0.3; 1.0) [amauroisis-1.0]	0.8 (0.5; 1.0) [0.001–1.0] 0.9 (0.6; 1.0) [0.001–1.0] 0.9 (0.6; 1.0) [0.001–1.0]	0.883^ g^ 0.518^ g^ 0.219^ g^	1.0 (0.7; 1.0) [0.01–1.0]	0.5 (0.02; 0.9) [amaurosis–1.0]	< 0.001^ g^
Longest diameter in mm^e^, median (25th; 75th percentiles) [range]	1.0 (0.7; 1.6) [0.1–8.0]	1.0 (0.8; 1.9) [0.2–8.0] 1.0 (0.8; 2.0) [0.2–8.0] 1.0 (0.6; 1.9) [0.2–8.0]	1.0 (0.6; 1.3) [0.1–2.4] 1.0 (0.5; 1.3) [0.1–4.0] 1.0 (0.7; 1.5) [0.1–6.0]	0.100^ g^ 0.06^ g^ 0.425^ g^	1.0 (0.5; 1.0) [0.1–4.1]	1.7 (1.3; 2.8) [0.5–8.0]	< 0.001^ g^
Absolute abundance of bacteria^f^, median (25th; 75th percentiles) [range]	232 (146.5; 452) [53–249873]	283 (152; 518) [64–249873] 310 (162, 536) [68–249873] 340 (162; 716) [64–249873]	192 (109; 288) [53–1821] 192 (192; 288 [53–1921] 190 (112; 310) [53–1821]	0.038^ g^ 0.005^ g^ 0.006^ g^	232 (135; 421) [53–44037]	233 (168; 756) [75–249873]	0.178^ g^

Visual acuity and lesion size are rounded up to one decimal. Hand motion = 0.001; finger counting = 0.01 [21]. All other figures are rounded up to the nearest whole number

^a^Refractive contact lens use (*n* = 64), aphakia correction (*n* = 1), bandage lens use (*n* = 3), and stable contact lens use (*n* = 1; this patient had undergone prior penetrating keratoplasty due to keratoconus)

^b^No contact lens wear: ocular surface disease (*n* = 11), prior ocular surgery (*n* = 4), trauma (*n* = 3), and no identified risk factor (*n* = 7)

^c^Information on symptom duration was missing for three patients

^d^Information on visual acuity was missing for five patients

^e^Information on lesion size was missing for one patient

^f^qPCR values were not available for five samples

^g^Mann-Whitney *U* test

^h^Chi-squared test

^i^Fisher’s exact test

### Culture results

Positive corneal cultures were detected in 70/94 (74%) of the episodes of microbial keratitis. Of these, 16 patients were culture-positive by standard culture only, 11 were culture-positive by ESwab culture only, and the remaining 43 were culture-positive by both methods. Monomicrobial growth was noted in 25 of the culture-positive patients and polymicrobial growth of 2–6 different bacteria belonging to 1–5 genera was seen in the remaining 45.

In the overall cohort, 151 different bacteria were isolated and identified to species level by culture (standard culture, ESwab culture, or both). Due to polymicrobial growth of different species belonging to the same genera among 13 patients, this corresponded at genus level to 127 distinct bacterial isolates belonging to 15 different bacterial genera: *Staphylococcus* (*n* = 46), *Streptococcus* (*n* = 2), *Corynebacterium* (*n* = 22), *Micrococcus* (*n* = 2), *Nocardia* (*n* = 1), *Brachybacterium* (*n* = 1), *Haemophilus* (*n* = 2), *Moraxella* (*n* = 3), *Enterococcus* (*n* = 3), *Enterobacter* (*n* = 1), *Pantoea* (*n* = 2), *Pseudomonas* (*n* = 3), *Serratia* (*n* = 1), *Cutibacterium* (*n* = 37), and *Veionella* (*n* = 1). An additional Gram-positive rod where no further identification was performed was also reported. Gram-positive bacteria constituted the largest group of isolated bacteria, predominantly *Staphylococcus* (46/127; 36%) and *Corynebacterium* (22/127; 17%). There were no dominant genera among the Gram-negative bacteria. Supplementary Table 3 contains information on bacteria isolated by the two culture methods.

### Prediction model for culture outcome of indirectly inoculated corneal samples (ESwab culture)

We used qPCR data from the ESwab samples to create a model for predicting a positive culture outcome from the same ESwab sample, including five independent variables: age, 16S rRNA copy number (log_10_units), prior antibiotic treatment, laterality, and sex. The model was statistically significant (*χ*^2^ (df 5) = 23.898; *p* < 0.001), explained 31.6% (Nagelkerke *R*^2^) of the variance in culture outcome, and correctly classified 67.4% of the cases with a sensitivity of 74.0% and specificity of 59.0%. Positive and negative predictive values were 69.8% and 63.9% respectively. Three of the five predictive variables were statistically significant: age, 16S rRNA gene copy number (log_10_units), and antibiotic treatment prior to sampling, Supplementary Table 4 summarizes the regression model. The 16S rRNA gene copy number (log_10_units) displayed an OR of 6.3 (95% CI, 1.6–25.0; *p* = 0.009). The odds for a positive culture outcome from the ESwab sample also increased with increasing age (OR 1.039; *p* = 0.034), while prior antibiotic treatment decreased the odds for a positive culture (OR 0.177; *p* = 0.031) (Supplementary table 4).

### Variables influencing the bacterial densities in the ESwab samples of patients with microbial keratitis

The 16S rRNA gene copy numbers displayed large dispersion within the cohort, with a range of 53–249,873 copies among the studied episodes of microbial keratitis (median copy number: 232). Univariate analysis revealed a significantly higher median copy number among the culture-positive patients compared to their culture-negative counterparts, regardless of culture method (standard culture, ESwab culture, or when all culture results were considered; Table 1). In the multivariate regression model with the five independent variables: lesion size (longest diameter), age, prior topical antibiotic treatment, risk factor for keratitis (in terms of contact lens wear or not), and sampling order (first or second) only lesion size had a significant influence on 16S rRNA gene copy number, with an increase of 65 copies for each mm increase in lesion diameter, Supplementary Table 5 summarizes the regression model.

### Microbiome profiles

**Fig. 1:**
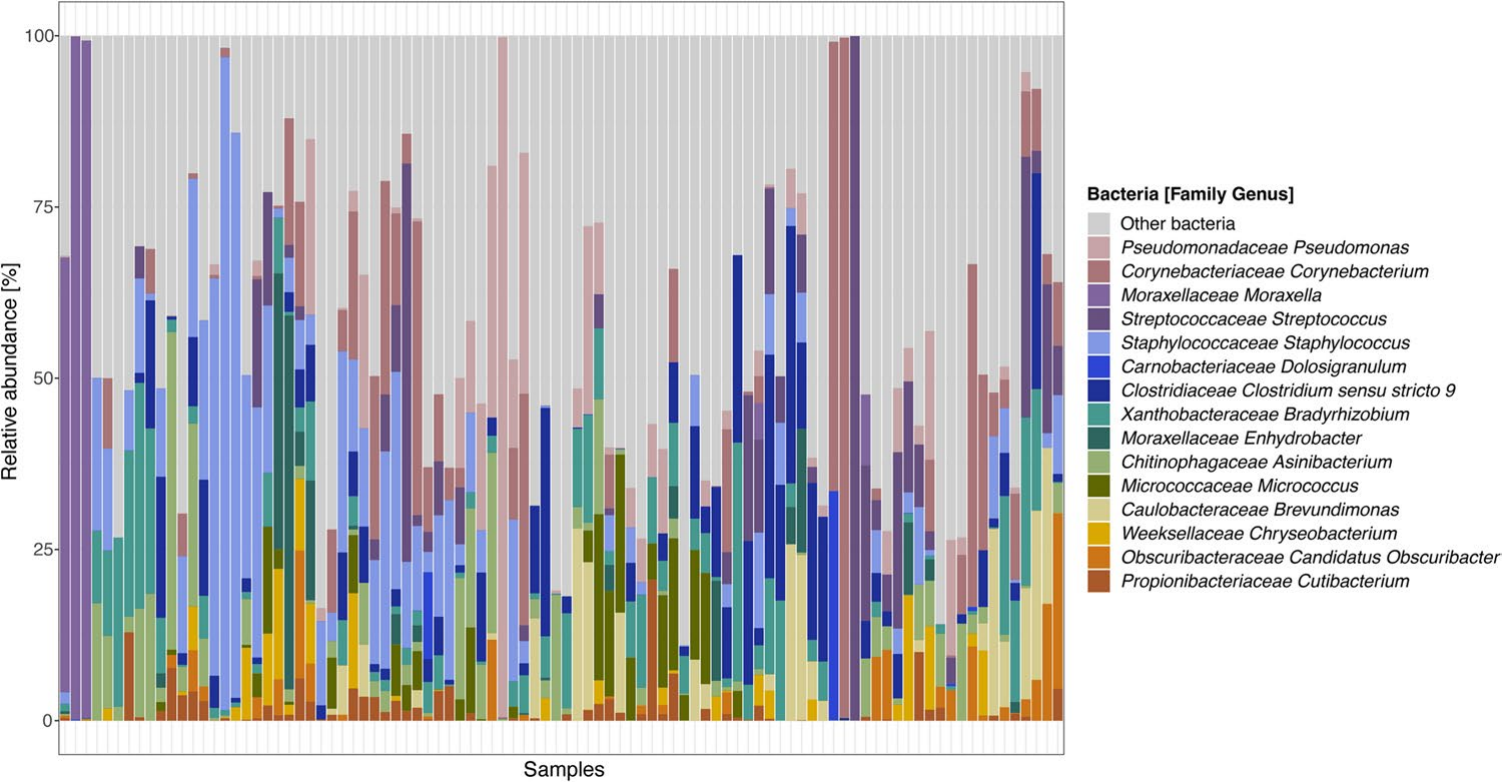
Bacterial communities within corneal samples of patients with microbial keratitis. Relative abundances (%) of the overall 15 most abundant bacterial genera (colored bars) and of the remaining identified bacterial genera (grey bars)

16S rRNA amplicon sequencing of the corneal samples revealed a median presence of 17 ASVs (range, 8–47) and 15 different bacterial genera (range, 8–30) per sample. Some samples were dominated by a single genus (e.g., *Staphylococcus*, *Pseudomonas Moraxella*, *Corynebacterium*, or *Streptococcus*), whereas others were more diverse and heterogeneous (Fig. 1). However, the ASV richness (alpha diversity) was not significantly influenced by disease severity in terms of duration of topical pharmacological treatment or surgical intervention (data not shown). No sample was PCR negative.

Overall, 13 of the 15 genera identified by culture methods could be detected by sequencing. At genus level, 96/127 (76%) of the different bacteria isolates detected by culture were also detected by sequencing within the same sample. From a patient perspective, this corresponds to a partial agreement rate of 87% between culture and targeted sequencing; that is, in 61/70 (87%) of the culture-positive patients, at least one bacterial genus that was isolated by culture was also detected by the sequencing approach. The complete agreement rate was 61%; in that 43/70 (61%) of the culture-positive patients had all of the genera isolated by culture also detected by targeted sequencing. Of the 24 culture-negative episodes, all were PCR positive, see below.

No ASVs belonging to either *Nocardi*a or *Pantoea* were detected by sequencing. The ASV richness (number of unique ASVs observed within a sample) was significantly higher within the culture-positive samples (median, 19 ASVs; interquartile range (IQR), 15–25) compared to the culture-negative samples (median, 15 ASVs; IQR, 13–17; *p* = 0.002).

**Fig. 2:**
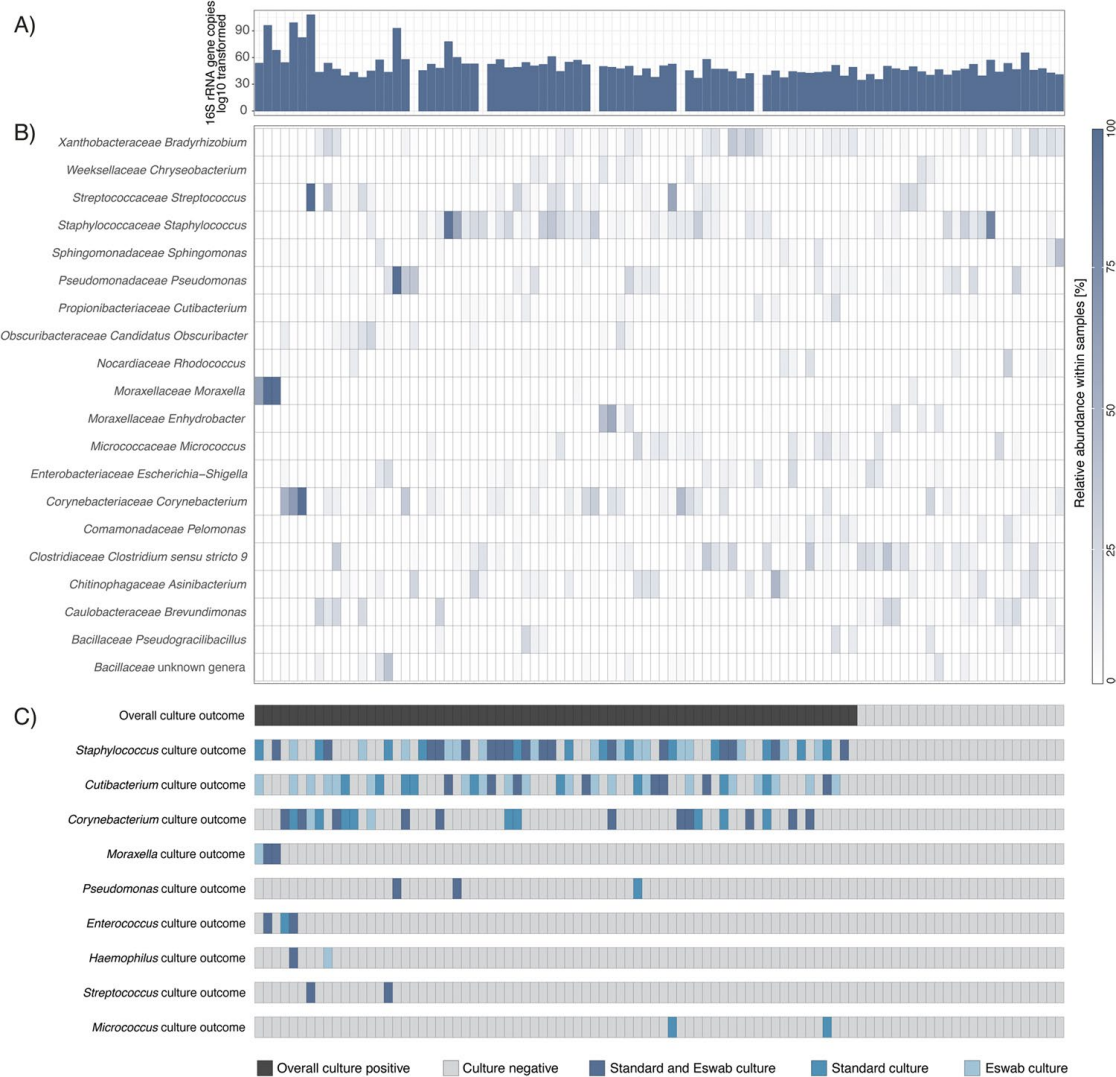
Comparison of targeted sequencing results and culture results. Each column represents a unique sample, organized according to culture outcome and bacterial community similarities. **A** Bar plot showing 16S rRNA gene copy numbers (log_10_ transformed counts) were measured by qPCR. Data were unavailable for five samples. **B** Heatmap showing the relative abundance of the overall 20 most abundant genera within the samples. **C** Tile plots showing the culture status for each sample. The first plot shows whether the samples were culture-positive for any bacteria (dark grey color) or not (light grey). The remaining plots show whether the samples were culture-positive for a specific organism, and if that organism was identified by standard culture, ESwab culture, or both (blue colors)

Among 9 of the 24 culture negative samples a previously described corneal pathogen [8, 22] was detected in the highest proportional abundance in the sample, in a proportional abundance of 22%-83% (Fig. 2A–C).

Of these nine patients, six received initial therapy with a fluoroquinolone alone and their samples displayed highest proportional abundance of *Pseudomonas* (*n* = 2), *Staphylococcus* (*n* = 2) and one each of *Veillonella* and *Clostridium*, respectively. A single patient received initial treatment of fortified drops i.e., vancomycin and ceftazidime, in this patient’s sample sequencing detected an 83% abundance of *Staphylococcus.* The remaining two patients were initially treated with a combination of fluoroquinolone and chloramphenicol, their samples displayed the highest proportional abundance of *Staphylococcus* and *Brevundimonas*, respectively. Only one patient, with the highest proportional abundance of *Pseudomonas*, received additional antibacterial therapy, with tobramycin due to clinical course, and only one patient, the one with initial fortified drops required surgical intervention with amniotic membrane. The total treatment duration (median; range) for respective treatment group was 17 days; 6–18 and 17, 5 days; 15–20; and 16 days for initial, empirical treatment with fluoroquinolone only, combination of fluoroquinolone, and chloramphenicol and fortified drops respectively.

Differential abundance analysis indicated that only *Brevundimonas* was significantly enriched within the culture-negative samples compared to the culture-positive samples (fold-change, 8.2; 95% CI, 2.1–31.3; adjusted *p* < 0.05).

**Fig. 3:**
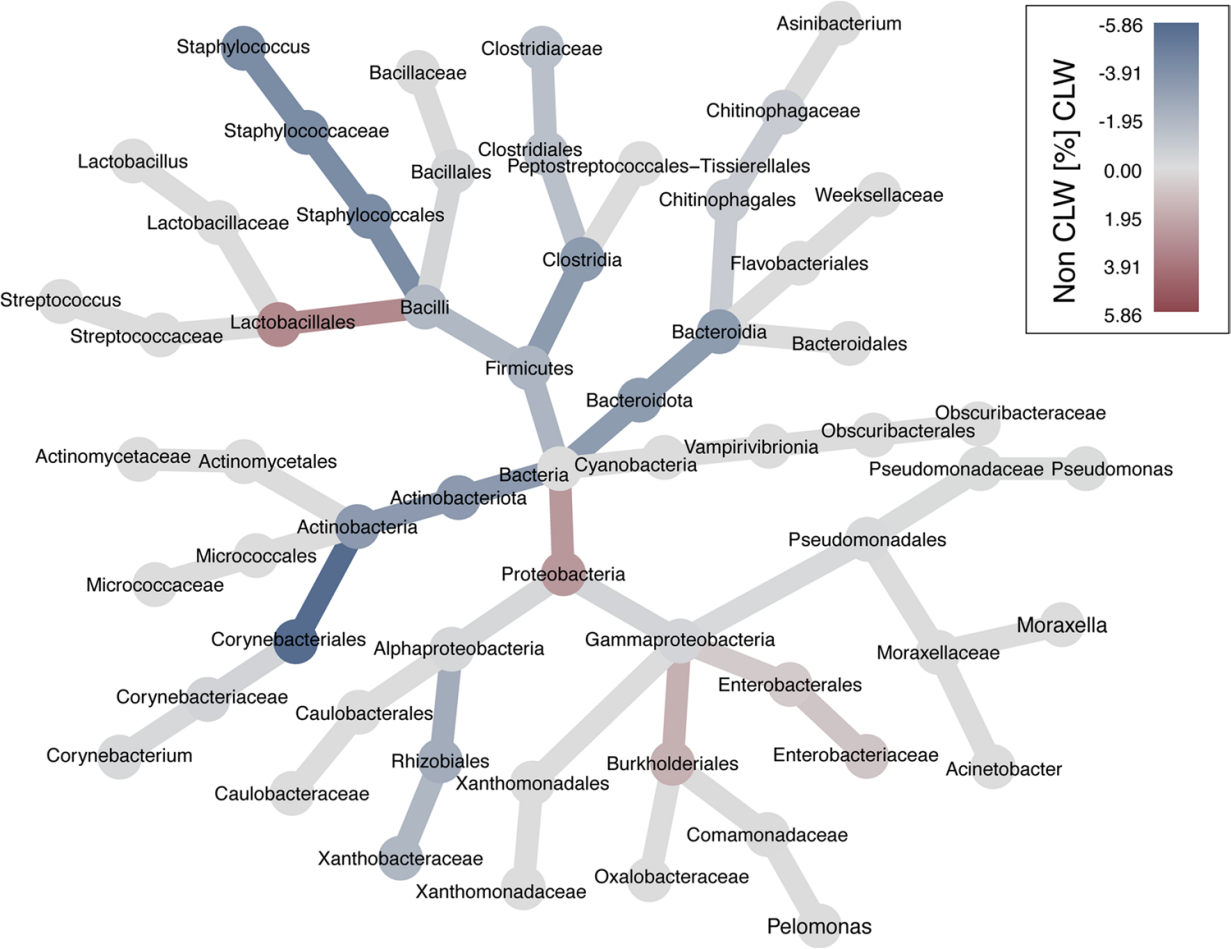
Bacterial taxa abundances in corneal samples from patients with microbial keratitis related to the presence or absence of contact lens wear. The heat tree includes bacterial taxa present in a minimum of 10 samples. Grey taxa indicate similar relative abundance between samples collected from contact lens wearers (CLW) and non-wearers (non CLW). Blue taxa were relatively more abundant in samples from contact lens wearers, and red taxa were relatively more abundant in samples from non-wearers

Bacterial compositional differences (beta diversity) (*R* = 0.14, *p* = 0.01) was observed between contact lens wearers and non-contact lens wearers, with communities being more homogeneous among the contact lens wearers. Part of this observed variation was likely due to higher proportional abundances of *Staphylococcus* spp. (fold-change: 11.7; 95% CI, 3.7–37.4; adjusted *p* < 0.01), *Corynebacterium* spp. (fold-change, 3.6; 95% CI, 1.6–7.8; adjusted *p* < 0.05), and *Stenotrophomonas maltophilia* (fold-change, 3.0; 95% CI, 1.8–5.3; adjusted *p* < 0.001) within the corneal lesions of contact lens wearers compared to non-wearers (Fig. 3). *S. maltophilia* was present in 13/69 (19%) of the samples collected from contact lens wearers, constituting more than 20% of the sequence reads in one of the patients, but was only detected in one sample among the 25 non-wearers, at a proportional abundance below 0.1%. There was no difference in the median ASV richness between the contact lens wearers and non-wearers.

Prior antibiotic treatment had no significant influence on the composition of bacterial communities in the corneal ulcer, as the composition (beta diversity) was similar among patients who were and were not treated with antibiotics prior to sampling. However, a reduced ASV richness (alpha diversity) was observed among the antibiotic-treated patients (median, 14 ASVs; IQR, 12–17) compared to the treatment-naïve (median, 18 ASVs; IQR, 14–22; *p* = 0.05). There was also a significant difference in the total number of unique genera when comparing antibiotic-treated patients (median, 13 genera; IQR, 10–15) with the treatment-naïve (median, 16 genera; IQR, 12–18; *p* = 0.02).

## Discussion

Microbial keratitis is an ophthalmological emergency and a common cause of corneal visual impairment and monocular blindness globally [23–25]. Corneal culture in cases of microbial keratitis has its limitations; for example, the median culture positivity rate is ≈50% of clinically cases of microbial keratitis [1]. In the literature, the clinical importance to identify the disease-causing pathogen is inconclusive [26–29].

In the present study, nine patients with a negative corneal culture displayed a previously reported corneal pathogen, in the highest abundance. Of these patients, six received topical empirical treatment with a fluoroquinolone only. Among those six patients, five displayed a corneal pathogen with a previously reported reduced sensitivity of fluoroquinolones of 20–38.8% [1, 30] One of these episodes required additional topical therapy, but none of these six patients required any surgical intervention. This favorable outcome may reflect the low prevalence of reduced susceptibility to fluoroquinolones reported previously from the setting of this present study [31].

In Europe, the USA, and Australia, episodes of microbial keratitis are dominated by a bacterial etiology [1], and an association with contact lens wear has been reported in 28.9–63% of cases [32–34]. In the present study, the rate of contact lens wear among patients with episodes of microbial keratitis was high, at 73%. This may be due to an awareness among contact lens wearers, opticians, and the healthcare system that unilateral redness and pain in combination with contact lens wear is enough to raise suspicion of microbial keratitis and should result in immediate contact with an ophthalmologist or equivalent. This in turn may also provide a partial explanation for the significantly shorter symptom duration, significantly higher visual acuity, and smaller lesions among the contact lens wearers in the present study, compared to those with other risk factors for microbial keratitis.

A majority of the genera isolated by culture in the cohort of patients with microbial keratitis could also be detected by applying 16S rRNA amplicon sequencing. In all, 76% of the different bacterial isolates identified at genus level were also detected by sequencing in the same sample. This is similar to previously reported concordance rates of 63–80% between sequencing and culture among patients with monomicrobial bacterial ulcers [5, 35]. In the present study, the microbiota of the corneal ulcers from the contact lens wearers were more homogenous than those of the ulcers from non-wearers, with higher proportional abundance of genera including *Staphylococcus* and *Corynebacterium*. This is in contrast to a previous report from the healthy conjunctiva [36].

We found that *S. maltophilia* was present in a significantly higher proportional abundance among the contact lens wearers than among patients with other risk factors for keratitis. *S*. *maltophilia* is an aerobic Gram-negative rod of the family *Xanthomonadaceae*. It is closely related to *Pseudomonas*, that can be found in the environment in water or humid settings, and is considered an opportunistic human pathogen with multidrug resistance potential [37, 38]. Trauma, contact lens wear, and penetrating keratoplasty have previously been reported as common risk factors among episodes of keratitis displaying growth of *S*. *maltophilia* [39, 40]. One explanation for the significantly higher abundance of *S. maltophilia* among contact lens wearers in the present study may be its ability to form biofilm. Both the contact lens itself and the lens case provide potential surfaces for biofilm growth, which gives a partial explanation for why contact lens wear is advantageous to this pathogen. This may be supported by the findings of Wiley et al., who reported that *S. maltophilia* is resistant to lens cleanser and was one of the dominant bacteria in biofilms detected on contact lenses [41].

In the present study, antibiotic treatment prior to sampling had no significant influence on either the composition of bacterial genera or the absolute number of bacteria (measured by qPCR) in the corneal ulcers of patients with microbial keratitis. This is supported by earlier finding by Darden et al. on the composition of the feline ocular surface microbiome after topical antibiotic treatment [42]. However, a reduced ASV richness was observed among the antibiotic-treated patients in the present study.

*Brevundimonas* was found to be significantly enriched among our culture-negative samples compared to the culture-positive samples. Other known corneal pathogens such as *Clostridium, Staphylococcus*, *Veillonella*, and *Pseudomonas* constituted at least 20% of the relative abundance among almost half of all of the culture-negative samples.

*Brevundimonas* spp. are Gram-negative, non-fermenting aerobe rods. Microbial keratitis presumably caused by both *Brevundimonas diminuta* [43] and *Brevundimonas vesicularis* [44] has been previously reported. In the case report of the *B. diminuta* keratitis, the bacteria was isolated from broth only, after at least seven days of incubation. Unfortunately, the culture media and incubation conditions were not included in the case report on the *B. vesicularis* keratitis. However, both *B. diminuta* and *B. vesicularis* are known to grow slowly on commonly-used nutrient media, but once isolated, can be correctly identified to species level with MALDI-TOF MS [45]. The lack of *Brevundimonas* isolated by culture in the present study may be due to the culture media and incubation conditions, since the fastidious anaerobe broth was discarded after seven days and no selective medium for Gram-negative bacteria was used. The findings of *Brevundimonas* by targeted sequencing may provide a potential causative pathogen among some of the culture-negative cases of microbial keratitis in the present study, as *Brevundimonas* constituted more than 10% of the reads in 5/24 samples.

The inoculation strategy (i.e., whether to transfer the corneal sample directly on culture media or indirectly via transport media) has been studied previously, but with inconclusive results [8, 46–49]. In the present study, we could explain > 30% of the variance in culture outcome of indirectly inoculated corneal samples and that the OR of a positive culture outcome increased with the 16S rRNA gene copy number and with age, and decreased with prior topical antibiotic treatment. At present, there is a lack of reports on variables influencing the culture outcome of indirectly inoculated corneal samples from patients with microbial keratitis [50–52].

The diagnostic and clinical applications of using qPCR to determine the absolute amount of bacteria in corneal samples of patients with microbial keratitis have been explored previously [4]. In the present study, the amount of bacteria displayed great variability (range of 53–249,873 16S rRNA gene copies) within the cohort, despite the prospective study design, strict inclusion criteria, and randomized sampling order with standardized sampling. We created a regression model in an attempt to explain this variance, but the model could only explain 0.3% of the variance in 16S rRNA gene copy number. Lesion size was the only variable with a significant effect on the copy number, highlighting the complex nature of the ocular microbiome.

Topical anesthetics have previously been reported to influence the microbiome findings from the conjunctiva [53]. This is likely due to patients’ tolerance of the pressure applied when sampling, since it has been reported that deep sampling (high pressure applied with dry swab) significantly affects the abundances of detected bacteria compared to soft sampling (minimal pressure applied with moist swab) [54]. In the present study, all patients received the same topical preservation-free anesthetics prior to sampling, the swab used for sampling in all ulcers was applied dry and was of the same model and fabrication in all patients, and samples were collected from a corneal ulcer rather than the conjunctiva. We did not standardize or control the duration or strength of pressure with which the sampling swab was applied to the corneal lesion. This may provide at least a partial explanation for the wide range of microbial DNA detected from the corneal lesions of our participants, and the difficulties in explaining this with the regression model used. There are likely also other factors, related both to the sampling process, clinical history, and patient characteristics and to the microbial characteristics and differences in pathogenicity, that influence the amount of bacterial DNA that can be detected from the corneal lesions of patients with microbial keratitis.

The strengths of the current study are the prospective design and the sample size. The main limitations are the lack of control of factors influencing the microbial DNA retrieval from the corneal ulcer, and the fact that only one sample per episode was retrieved for sequencing.

In conclusion, in 87% of the culture-positive episodes of microbial keratitis, sequencing detected at least one of the bacterial genera isolated by culture. Among more than one third of the culture-negative episodes, sequencing detected a previously described corneal pathogen in the highest proportional abundance. These findings indicate that targeted sequencing may provide valuable information in a clinical context. However, future studies, which also allow for information from molecular diagnostic methods, are needed to evaluate if this additional information significantly improves the outcome for patients with microbial keratitis, and/or provides other health economic benefits. Corneal lesions among contact lens wearers displayed a significantly different bacterial community composition compared to lesions of patients with other risk factors for microbial keratitis. Finally, quantitative data from targeted sequencing indicated that the culture outcome of indirectly inoculated corneal samples may be influenced by the absolute bacteria count in the sample.

### Supplementary Information

Below is the link to the electronic supplementary material.

Supplementary file1 (PDF 62.3 KB)

Supplementary file2 (PDF 289 KB)

Supplementary file3 (PDF 275 KB)

Supplementary file4 (PDF 118 KB)

Supplementary file5 (PDF 187 KB)

Supplementary file6 (PDF 185 KB)

Supplementary file7 (DOCX 25 KB)
