# Supplementary material for: In the eye of the ophthalmologist: the corneal microbiome in microbial keratitis
Source: Graefes Arch Clin Exp Ophthalmol. 2023 Nov 23;262(5):1579–89. doi: 10.1007/s00417-023-06310-y (PMC11031470; doi:10.1007/s00417-023-06310-y)
Supplement: Supplementary file 1 — Supplementary file1 (PDF 62.3 KB) [file 417_2023_6310_MOESM1_ESM.pdf]

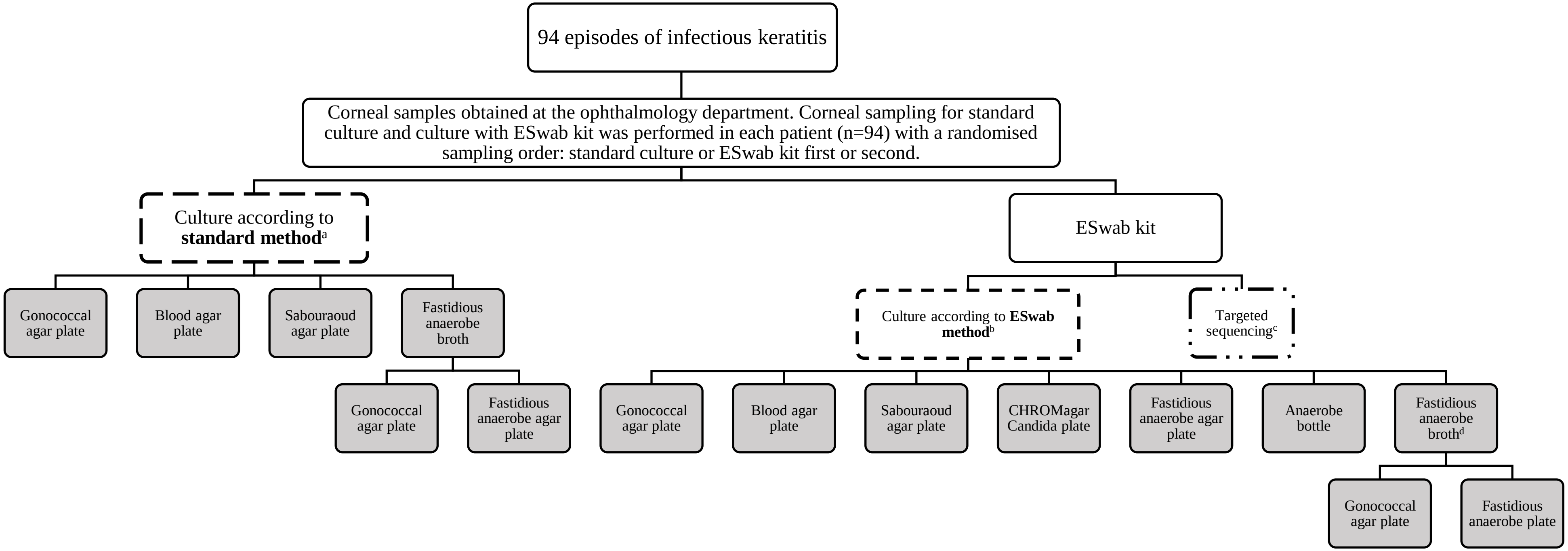

Supplementary Fig 1. Flowchart of the two different corneal culture approaches applied in the study.

[a] According to standard culture, the corneal samples were directly transferred to culture media at the time of sampling, i.e. at the ophthalmology department, and thereafter transported to the laboratory for incubation. [b] According to ESwab culture, the corneal sample was transported in the liquid amies transport medium of the ESwab kit to the laboratory where 30 µl was transferred to each of the solid agar plates and the fastidious anaerobe broth respectively and 200 µl was transferred to the anaerobe bottle. [c] Remaining transport medium with corneal sample was stored at -80 degrees C pending targeted PCR and sequencing. [d] Nine patients lacked culture results from Eswab sample passed through enrichment in FAB.
