# Supplementary material for: In the eye of the ophthalmologist: the corneal microbiome in microbial keratitis
Source: Graefes Arch Clin Exp Ophthalmol. 2023 Nov 23;262(5):1579–89. doi: 10.1007/s00417-023-06310-y (PMC11031470; doi:10.1007/s00417-023-06310-y)
Supplement: Supplementary file 2 — Supplementary file2 (PDF 289 KB) [file 417_2023_6310_MOESM2_ESM.pdf]

Supplementary Table 1 Culture media and incubation conditions for corneal culture, according to “standard culture” and “ESwab culture”, of the 94 episodes of microbial keratitis

**Culture media** [a]	**Contents/manufacturer**	**Incubation**
Gonococcal agar plate	GC Medium Base (Becton Dickinson, Sparks, MD, USA) supplemented with chocolatized defibrinated horse blood	5% CO_2 at 36°C
Blood agar plate	3.9% Columbia Blood Agar Base, (Oxoid, Basingstoke, Hampshire, UK) supplemented with 6% defibrinated horse blood	Day 1: air at 36°C<br>Days 2–7: CO_2 at 36°C
Sabouraud agar plate	1.3% Agar No 2 (Lab M, Heywood, Bury, UK), 4% D-Glucose (VWR, Leuven, Belgium), 1% Peptone (Becton Dickinson)	Air at 30°C
Fastidious anaerobe broth	2.97% FAB (Lab M) supplemented with 1% D-glucose (VWR)	Air at 36°C. Sub-cultured when growth seen or after 7 days
Fastidious anaerobe agar plate	4.6% (w/v) LAB 90 Fastidious Anaerobe Agar (Lab M) supplemented with 5% (v/v) horse blood	Anaerobic atmosphere (80% N_2, 10% CO_2, 10% H_2) at 36° C
CHROMagar Candida plate	4.77%(w/v(CHROMagar candida (CHROMagar, Paris, France)	Air at 36°C
Anaerobe bottle	Blood culture bottle BD Bactec systems standard anaerobic medium (Becton Dickinson, Franklin Lake, NJ, USA)	Incubator

[a] For the ESwab culture, 30 µl of the modified Amies transport medium of the ESwab kit was indirectly inoculated onto each of the solid plates and in the fastidious anaerobe broth, and an additional 200 µl was dispensed in the anaerobe bottle.
