# Supplementary material for: In the eye of the ophthalmologist: the corneal microbiome in microbial keratitis
Source: Graefes Arch Clin Exp Ophthalmol. 2023 Nov 23;262(5):1579–89. doi: 10.1007/s00417-023-06310-y (PMC11031470; doi:10.1007/s00417-023-06310-y)
Supplement: Supplementary file 3 — Supplementary file3 (PDF 275 KB) [file 417_2023_6310_MOESM3_ESM.pdf]

Supplementary Table 2 Topical antibiotics that preceded corneal sampling for culture and sequencing in 16 episodes of microbial keratitis

**Topical antibiotics**	**Patients (n=16)**
Fluoroquinolones	2
Chloramphenicol	6
Fusidic acid	2
Fluoroquinolones and chloramphenicol	2
Other [a]	4

[a] One patient was initially treated with a combination of fluoroquinolones and chloramphenicol that was later adjusted to vancomycin and ceftazidime, two patients received oxytetracycline in combination with hydrocortisone, and one patient was pretreated with a combination of fluoroquinolones, tobramycin, and chloramphenicol. Ten patients discontinued treatment 12–96 h prior to sampling, five did not, and for the remaining patient information on treatment continuation/discontinuation was missing.
