# Supplementary material for: In the eye of the ophthalmologist: the corneal microbiome in microbial keratitis
Source: Graefes Arch Clin Exp Ophthalmol. 2023 Nov 23;262(5):1579–89. doi: 10.1007/s00417-023-06310-y (PMC11031470; doi:10.1007/s00417-023-06310-y)
Supplement: Supplementary file 4 — Supplementary file4 (PDF 118 KB) [file 417_2023_6310_MOESM4_ESM.pdf]

Supplementary Table 3 Microorganisms isolated by standard culture, ESwab culture, or both

**Bacteria**	**Isolated by both standard and ESwab culture**	**Isolated by standard culture only**	**Isolated by indirect inoculation of ESwab sample only**
Staphylococcus aureus (n=6)	1	4	1
Coagulase-negative staphylococci (n=62)	14 [a]	22 [b]	26 [c]
Streptococcus pneumonia (n=1)	1	0	0
Streptococcus mitis (n=1)	1	0	0
Corynebacterium spp. (n=24)	11 [d]	10 [e]	3 [f]
Micrococcus spp. (n=1)	0	1	0
Micrococcus luteus (n=1)	0	1	0
Nocardia sp. (n=1)	1	0	0
Brachybacterium spp. (n=1)	0	1	0
Gram-positive rod (n=1)	0	0	1
**Total Gram-positive (n=99)**	**29**	**39**	**31**
Haemophilus parainfluenzae (n=2)	1	0	1
Moraxella nonliquefaciens (n=2)	1	0	1
Moraxella spp. (n=1)	1	0	0
Enterococcus faecalis (n=3)	2	1	0
Enterobacter cloacae (n=1)	0	0	1
Pantoea septica (n=2)	0	1	1
Pseudomonas aeruginosa (n=3)	2	1	0
Serratia marcescens (n=1)	0	0	1
**Total Gram-negative (n=15)**	**7**	**3**	**5**
Cutibacterium acnes (n=36)	8	9	19
Cutibacterium avidum (n=1)	0	0	1
Veionella parvula (n=1)	0	1	0
**Total anaerobes (n=38)**	**8**	**10**	**20**
**Total bacteria (n=152)**	**44**	**52**	**56**
Candida albicans (n=1)	0	1	0
Cladosporium spp. (n=1)	0	1	0

[a] *S. epidermidis* (n=9), *S. capitis* (n=3), *S. lugdunensis* (n=1), *S. warneri* (n=1). [b] *S .epidermidis* (n=18), *S. capitis* (n=3), *S. hominis* (n=1). [c] *S. epidermidis* (n=17), *S. capitis* (n=2), *S. lugdunensis* (n=2), *S. saccharolyticus* (n=1), *S. haemolyticus* (n=1), *S. simulans* (n=1), *S. warneri* (n=1), CoNS – no further identified (n=1). [d] *C. macginleyi* (n=8), *C. propinquum* (n=1), *C. mastitidis* (n=1), *Corynebacterium* spp. (n=1). [e] *C. macginleyi* (n=5), *C. tuberculostearicum* (n=1), *C. accolens* (n=1), *C. bovis* (n=1), *C. pseudodiphtheriticum* (n=1), *Corynebacterium* spp. (n=1). [f] *C. macginleyi* (n=2), *C. accolens* (n=1).
