# Supplementary material for: In the eye of the ophthalmologist: the corneal microbiome in microbial keratitis
Source: Graefes Arch Clin Exp Ophthalmol. 2023 Nov 23;262(5):1579–89. doi: 10.1007/s00417-023-06310-y (PMC11031470; doi:10.1007/s00417-023-06310-y)
Supplement: Supplementary file 5 — Supplementary file5 (PDF 187 KB) [file 417_2023_6310_MOESM5_ESM.pdf]

Supplementary Table 4 Multivariate analysis on variables influencing culture outcome of indirectly inoculated corneal samples of patients with microbial keratitis

| | **B** | **SE** | **Wald** | **df** | **Sig** | **Exp(B)** | **95% CI for Exp(B)** | |
|---|---|---|---|---|---|---|---|---|
| | | | | | | | **Lower** | **Upper** |
| Age | .039 | .018 | 4.488 | 1 | .034 | 1.039 | 1.003 | 1.077 |
| Male sex | -.806 | .523 | 2.376 | 1 | .123 | .446 | .160 | 1.245 |
| Topical antibiotics prior to sampling | -1.730 | .804 | 4.628 | 1 | .031 | .177 | .037 | .857 |
| Right eye | .954 | .499 | 3.656 | 1 | .056 | 2.597 | .976 | 6.906 |
| 16S rRNA gene copy number ($log_{10}$units) | 1.841 | .702 | 6.871 | 1 | .009 | 6.303 | 1.591 | 24.968 |
| Constant | 5.615 | 1.907 | 8.673 | 1 | .003 | .004 | | |

With one exception, all standardized residuals were within ±3. The Box-Tidwell test for a nonlinear relationship between the variables and the logit of culture outcome was not significant, no multicollinearity between the variables was detected, and the variance inflation factor for the five predictor variables ranged from 1.033 to 1.222.
