# Supplementary material for: In the eye of the ophthalmologist: the corneal microbiome in microbial keratitis
Source: Graefes Arch Clin Exp Ophthalmol. 2023 Nov 23;262(5):1579–89. doi: 10.1007/s00417-023-06310-y (PMC11031470; doi:10.1007/s00417-023-06310-y)
Supplement: Supplementary file 6 — Supplementary file6 (PDF 185 KB) [file 417_2023_6310_MOESM6_ESM.pdf]

Supplementary Table 5 Quantile multivariate analysis on variables influencing the absolute bacterial amount form corneal samples dispensed in liquid transport medium obtained from patients with microbial keratitis

**Parameter**	**Coefficient**	**Standard error**	**t**	**df**	**Sig**	**95% confidence interval**	
						**Lower bound**	**Upper bound**
(Intercept)	192.319	143.8300	1.337	82	.185	-93.804	478.443
Age at disease onset	.923	2.2218	.415	82	.679	-3.497	5.343
Lesion size (mm)	64.942	29.0781	2.233	82	.028	7.096	122.787
Sampling order (ESwab last)	-77.646	65.8855	-1.178	82	.242	-208.713	53.421
No topical antibiotics prior to sampling	-7.424	88.9931	-.083	82	.934	-184.460	169.611
No contact lens wear	-4.490	80.5326	-.056	82	.956	-164.695	155.716
