# Supplementary material for: In the eye of the ophthalmologist: the corneal microbiome in microbial keratitis
Source: Graefes Arch Clin Exp Ophthalmol. 2023 Nov 23;262(5):1579–89. doi: 10.1007/s00417-023-06310-y (PMC11031470; doi:10.1007/s00417-023-06310-y)
Supplement: Supplementary file 7 — Supplementary file7 (DOCX 25 KB) [file 417_2023_6310_MOESM7_ESM.docx]

**Supplementary methodology**

**Details on primers and on the PCR methodology**

*DNA extraction and 16S rRNA amplicon sequencing*

Lysozyme (3 mg/sample), lysostaphin (4 units/sample), and mutanolysin (25 units/sample) (Sigma-Aldrich, Merck, Germany) were added to 200 µl of samples and incubated at 37°C for 30 minutes followed by 30 minutes of incubation with protein kinase K (Sigma-Aldrich, Merck, Germany) at 56°C. DNA was then extracted from the samples on a MagNA Pure 96 instrument (Roche, Mannheim, Germany) using a DNA and Viral NA Small Volume Kit (Roche, Mannheim, Germany). A negative control sample (dH2O) and a positive control sample (bacterial mock community from ZymoBIOMICS; D6300, Zymo Research, CA, USA) were also included.

The following three sets of primers with heterogeneity spacers targeting the V3–V4 region of the 16SrRNA gene were used in the primary PCR. Illumina adaptor sequences are marked in blue.

forward 01: 5’-TCGTCGGCAGCGTCAGATGTGTATAAGAGACAGCCTACGGGNGGCWGCAG-3’

forward 02: 5’-TCGTCGGCAGCGTCAGATGTGTATAAGAGACAGGACCTACGGGNGGCWGCAG-3’

forward 03: 5’-TCGTCGGCAGCGTCAGATGTGTATAAGAGACAGTGAGACTCCTACGGGNGGCWGCAG-3’

reverse 01: 5’-GTCTCGTGGGCTCGGAGATGTGTATAAGAGACAGGACTACHVGGGTATCTAATCC-3’

reverse 02: 5’-GTCTCGTGGGCTCGGAGATGTGTATAAGAGACAGTCGACTACHVGGGTATCTAATCC-3’

reverse 03: 5’-GTCTCGTGGGCTCGGAGATGTGTATAAGAGACAGCTACTAGACTACHVGGGTATCTAATCC-3’

The PCR program for the initial amplification of the V3–V4 *16S rRNA* gene was as follows.

| Temp. | Time | Temp. | Time | Temp. | Time | Cycles |
| --- | --- | --- | --- | --- | --- | --- |
| 95°C | 3 min |  |  |  |  |  |
| 98°C | 20 sec | 60°C | 15 sec | 72°C | 45 sec | 25 |
| 72°C | 5 min |  |  |  |  |  |
| 4°C | ∞ |  |  |  |  |  |

Next, sample indexing using Nextera XT Index primers (kit v2 set A–D, Illumina Inc., San Diego, CA, USA) was performed in a secondary PCR, using the following program.

| Temp. | Time | Temp. | Time | Temp. | Time | Cycles |
| --- | --- | --- | --- | --- | --- | --- |
| 95°C | 3 min |  |  |  |  |  |
| 98°C | 20 sec | 55°C | 15 sec | 72°C | 45 sec | 20 |
| 72°C | 5 min |  |  |  |  |  |
| 4°C | ∞ |  |  |  |  |  |

Primers and primer dimer DNA were removed using AMPure XP (Beckman Coulter Inc., CA, USA) magnetic bead cleanup with a sample:bead volume ratio of 1:0.72. DNA concentration was measured (AccuClear Ultra High Sensitivity dsDNA Quantitation Kit, Biotium, CA, USA), and samples were pooled (equal DNA amount from each sample). Amplicon libraries (4nM DNA) were sequenced on a MiSeq instrument using an Illumina 600-cycle reagent kit V3.

*Quantitative PCR*

Primers targeting the same regions as for the amplicon sequencing were used together with an 16S-TQM-528R TaqMan™ probe (slightly modified from the process used by Nadkarni *et al*.[1] as follows.

Forward: 5’CCTACGGGNGGCWGCAG

Reverse: 5’GACTACHVGGGTATCTAATCC

Probe: 5´-FAM-CGTATTACCGCGGCTGCTGGCAC-BHQ-1

The master mix (primers, 2x PerfeCTa qPCR ToughMix Low ROX [Quanta BioSciences Inc., Gaithersburg, Maryland, USA], and 1 mM dithiothreitol) was treated with 0.4 U recombinant dsDNase (ArcticZymes, Tromsø, Norway) to eliminate potential PCR reagent bacterial DNA contaminants. The nuclease was activated by heating the mixture at 37°C for 20 min and subsequently inactivated by heating at 60°C for 20 min before the addition of template (2 µl). A negative control (dH_2_O) was included in the PCR.

The following PCR program was used for amplification.

| Temp. | Time | Temp. | Time | Temp. | Time | Cycles |
| --- | --- | --- | --- | --- | --- | --- |
| 95°C | 15 sec | 55°C | 30 sec | 72°C | 30sec | 50 |
|  |  |  |  |  |  |  |

Ten-fold dilutions of *Legionella pneumophila* purified genomic DNA ranging from 1000 to 10e6 genome equivalents per well were used to generate a standard curve, and the quantity of bacterial DNA in the samples was determined from the regression line.

*Sequence pre-processing*

Raw reads were demultiplexed with bcl2fastq Conversion Software (Illumina Inc., San Diego, CA, USA) and primer regions were trimmed off using Cutadapt (v.2.3) [2] at an 8% error rate (allowing one mismatch per primer) in paired-end mode. Trimmed reads were quality filtered, and amplicon sequence variants (ASVs) were inferred with DADA2 (v.1.12.1)[3] using default settings except for truncation length; forward reads were truncated at 270 bp and reverse reads at 210 bp. Consensus chimera were removed. Taxonomic assignment of ASVs was performed with DADA2’s assignTaxonomy and addSpecies functions, using the Silva reference database [4] and species-level training set (v.138) formatted for DADA2, respectively. ASVs not classified at order level (n=6) and ASVs belonging to chloroplasts (n=8) and mitochondria (n=8) were removed prior to analysis. Samples with fewer than 1000 reads were excluded, (n=2).[5]

**References**

1. Nadkarni MA, Martin FE, Jacques NA, et al (2002) Determination of bacterial load by real-time PCR using a broad-range (universal) probe and primers set. Microbiology (Reading) 148 (Pt 1):257-266. doi:10.1099/00221287-148-1-257

2. Martin M (2011) Cutadapt removes adapter sequences from high-throughput sequencing reads. 2011 17 (1):3. doi:10.14806/ej.17.1.200

3. Callahan BJ, McMurdie PJ, Rosen MJ, et al (2016) DADA2: High-resolution sample inference from Illumina amplicon data. Nat Methods 13 (7):581-583. doi:10.1038/nmeth.3869

4. McLaren MR (2020) Silva SSU taxonomic training data formatted for DADA2 (Silva version 138). Version2 edn. Zenodo. doi:10.5281/zenodo.3731176

5. Shivaji S, Jayasudha R, Chakravarthy SK, et al (2021) Alterations in the conjunctival surface bacterial microbiome in bacterial keratitis patients. Exp Eye Res 203:108418. doi:10.1016/j.exer.2020.108418
